# High intestinal carriage of *Clostridium perfringens* in healthy individuals and ICU patients in Hangzhou, China

**DOI:** 10.1128/spectrum.03385-23

**Published:** 2024-05-21

**Authors:** Zelin Yan, Bo Fu, Yanyan Zhu, Yanyan Zhang, Yuchen Wu, Panfeng Xiong, Hongwei Zhou, Yang Wang, Shaolin Wang, Gongxiang Chen, Rong Zhang, Chengtao Sun

**Affiliations:** 1 Department of Clinical Laboratory, School of Medicine, Second Affiliated Hospital of Zhejiang University, Hangzhou, China; 2 National Key Laboratory of Veterinary Public Health and Safety, College of Veterinary Medicine, China Agricultural University, Beijing, China; 3 Key Laboratory of Applied Technology on Green-Eco-Healthy Animal Husbandry of Zhejiang Province, Zhejiang Provincial Engineering Research Center for Animal Health Diagnostics and Advanced Technology, Zhejiang International Science and Technology Cooperation Base for Veterinary Medicine and Health Management, Zhejiang Agricultural and Forestry University, Hangzhou, China; University of Nebraska-Lincoln, Lincoln, Nebraska, USA

**Keywords:** *Clostridium perfringens*, clinical setting, antimicrobial resistance, toxinotype

## Abstract

*Clostridium perfringens* has emerged as a growing public health concern due to its ability to cause various infections and its increasing resistance to antibiotics. To assess its current epidemiology in clinical settings, we conducted a survey involving 426 healthy individuals and 273 ICU inpatients at a provincial hospital in China. Our findings revealed a high prevalence of *C. perfringens* in healthy individuals (45.77%, 95% CI: 41.0%–50.6%) and ICU patients (12.82%, 95% CI: 9.1%–17.4%). The identified 220 *C*. *perfringens* isolates displayed substantial resistance to erythromycin (57.9%), clindamycin (50.7%), and tetracycline (32.0%), primarily attributed to the presence of *erm*(Q) (54.4%), *lnu*(P) (13.8%), *tet*B(P) (83.6%), and *tet*A(P) (66.7%). Notably, *C. perfringens* isolates from this particular hospital demonstrated a high degree of sequence type diversity and phylogenic variation, suggesting that the potential risk of infection primarily arises from the bacteria’s gut colonization rather than clonal transmissions within the clinical environment. This study provides an updated analysis of the current epidemiology of *C. perfringens* in healthy individuals and ICU patients in China and emphasizes the need to optimize intervention strategies against its public health threat.

**IMPORTANCE:**

*Clostridium perfringens* is a bacterium of growing public health concern due to its ability to cause infections and its increasing resistance to antibiotics. Understanding its epidemiology in clinical settings is essential for intervention strategies. This study surveyed healthy individuals and ICU inpatients in a provincial hospital in China. It found a high prevalence of *C. perfringens*, indicating infection risk. The isolates also showed significant antibiotic resistance. Importantly, the study revealed diverse sequence types and phylogenetic variation, suggesting infection risk from intestinal colonization rather than clonal transmission in hospitals. This analysis emphasizes the need to optimize intervention strategies against this public health threat.

## INTRODUCTION

*Clostridium perfringens*, a Gram-positive, anaerobic, spore-forming bacterium, is ubiquitously found in soil, animal, and human intestines and feces. Under specific circumstances, this widely dispersed microorganism emerges as a prevalent pathogen, causing a spectrum of enteric or non-enteric diseases in both humans and animals. These include histotoxic and enteric infections, foodborne illnesses, non-food-related diarrhea, and enterocolitis (1). *C. perfringens* strains are notorious for their pathophysiology and virulence, attributable to the activity of over 20 identified toxins or enzymes (2). Based on the presence of six key toxins, seven distinct toxinotypes have been classified, enabling the differentiation of strains with potential associations with severe infectious outcomes (3).

In comparison to commonly encountered commensal bacteria such as *Klebsiella pneumoniae* of the Enterobacteriaceae family (4) and *Enterococcus* spp. of the *Enterococcaceae* families (5), *C. perfringens* nowadays exhibits a relatively narrow range of antibiotic resistance. Studies have reported that *C. perfringens* remains sensitive to a range of commonly used antimicrobial agents in human clinical practice (6). This intriguing observation can be attributed, at least in part, to the relatively infrequent carriage of plasmids by *C. perfringens* (7), thereby posing significant challenges in the acquisition of antibiotic resistance mechanisms. Nonetheless, over recent decades, the extensive use of antimicrobial agents in human clinical and animal settings has still contributed to the emergence of antibiotic resistance in *C. perfringens*. Widespread resistance to tetracycline, macrolide, and lincosamide, mediated by the *tet* and *erm* determinants, has been reported in *C. perfringens* isolates originating from both animals and human clinical settings (8, 9). Additionally, the emergence of the multi-resistance phenotype, PhLOPS_A_, mediated by the *cfr*(C) gene, has been identified in *C. perfringens* isolates from cattle in China (10), as well as various *Clostridium* spp. isolates (11, 12). These findings underscore the emerging risk of *Clostridium* spp. in acquiring and disseminating resistance determinants under the selection pressure of antimicrobial agents.

Within the clinical settings, effective intervention strategies to control the spread and infections caused by *C. perfringens* rely on a comprehensive understanding of its current epidemiology. However, the surveillance of *C. perfringens* in humans is not a routine practice, and its epidemiology remains largely unknown. Considering the clinical importance of its pathophysiology and the increasing resistance trends, increased surveillance is recommended to assess the current situation of *C. perfringens* (13). In this study, we conducted a cross-sectional investigation of *C. perfringens* in both healthy individuals and ICU patients at a provincial hospital in China. Our findings revealed the prevalence of *C. perfringens* in these populations, along with a high level of genomic diversity.

## RESULTS

### *C. perfringens* is prevalent in the surveyed population

From 22 October 2022 to 28 November 2022, we collected 699 stools and rectal swabs from 426 healthy subjects and 273 ICU inpatients at the Second Affiliated Hospital of Zhejiang University College of Medicine, Hangzhou, China. A total of 220 (31.47%, 95% CI: 28.0%–35.1%) *C. perfringens* isolates were obtained from the samples. In this cross-sectional study, we found a higher prevalence of *C. perfringens* in healthy individuals (195/426, 45.77%, 95% CI: 41.0%–50.6%) compared to ICU patients (35/273, 12.82%, 95% CI: 9.1%–17.4%) (*P* < 0.001). To facilitate further analysis, genomes from all 220 isolates were sequenced. Quality control procedures resulted in the acquisition of 195 strains from both healthy individuals (*n* = 36) and ICU patients (*n* = 159).

### Antimicrobial susceptibility profiles

**Fig 1:**
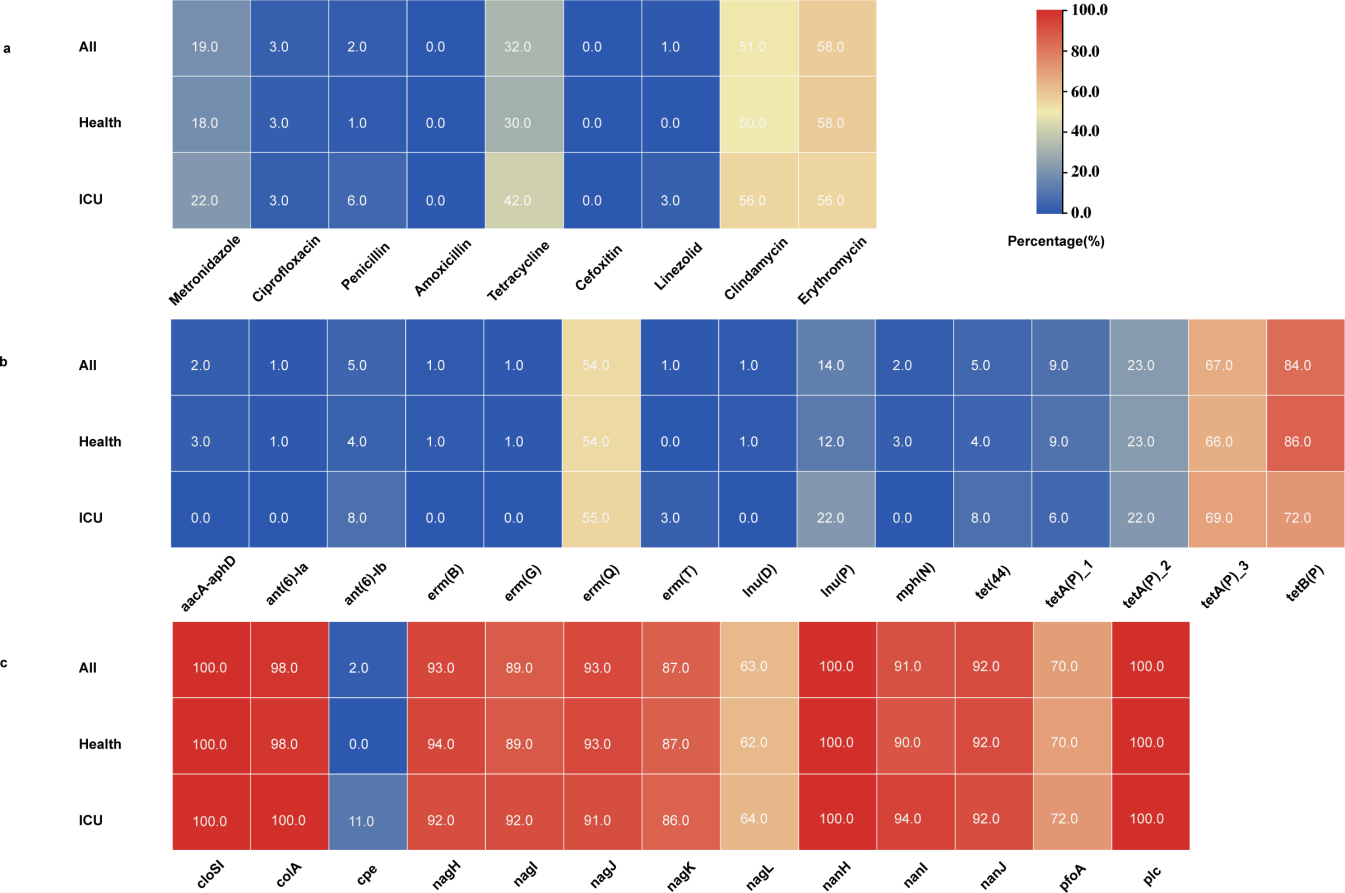
Percentage of *C. perfringens* resistant to a particular antimicrobial agent (**a**), carrying a particular resistance gene (**b**) or key virulence gene (**c**).

To guide a better clinical antibiotic treatment for *C. perfringens* infections, we conducted Etest on the isolated 220 *C*. *perfringens* isolates against nine antibiotics that are commonly used in *C. perfringens* infections. The tested *C. perfringens* isolates exhibited high resistance rates (over 50.0%) to erythromycin (57.9%) and clindamycin (50.7%) (Fig. 1a) and moderate resistance to tetracycline (32.0%); this was in accordance with the high presence of resistance genes in their genomes, including the erythromycin resistance gene *erm*(Q) (54.4%), lincosamide resistance gene *lnu*(P) (13.8%), and tetracycline resistance genes *tet*B(P) (83.6%) and tetA(P) (66.7%) (Fig. 1b). Plasmid detection, as measured by the presence of plasmid *rep* genes, showed that only 3 of 195 strains contained plasmids, indicating that resistance genes are mainly chromosomally borne. In contrast, the isolates were generally sensitive (resistance rate, below 5.0%) to penicillin, amoxicillin, cefoxitin, ciprofloxacin, and linezolid. Notably, one strain exhibited a high minimum inhibitory concentration (MIC) to linezolid (8 µg/mL). However, a search for mutations and acquired resistance genes encoding linezolid resistance in its genome revealed no known determinants. Overall, there was no significant difference in resistance rates to the tested antibiotics between *C. perfringens* from healthy individuals and ICU patients (*P* = 0.13). In addition, the acquired antimicrobial resistance genes were commonly found in both healthy individuals and ICU patients [3.00 (IQR: 2.00–3.00) vs 3 (IQR: 2.0–3.75); *P* = 0.773].

### High genomic diversity of *C. perfringens*

**Fig 2:**
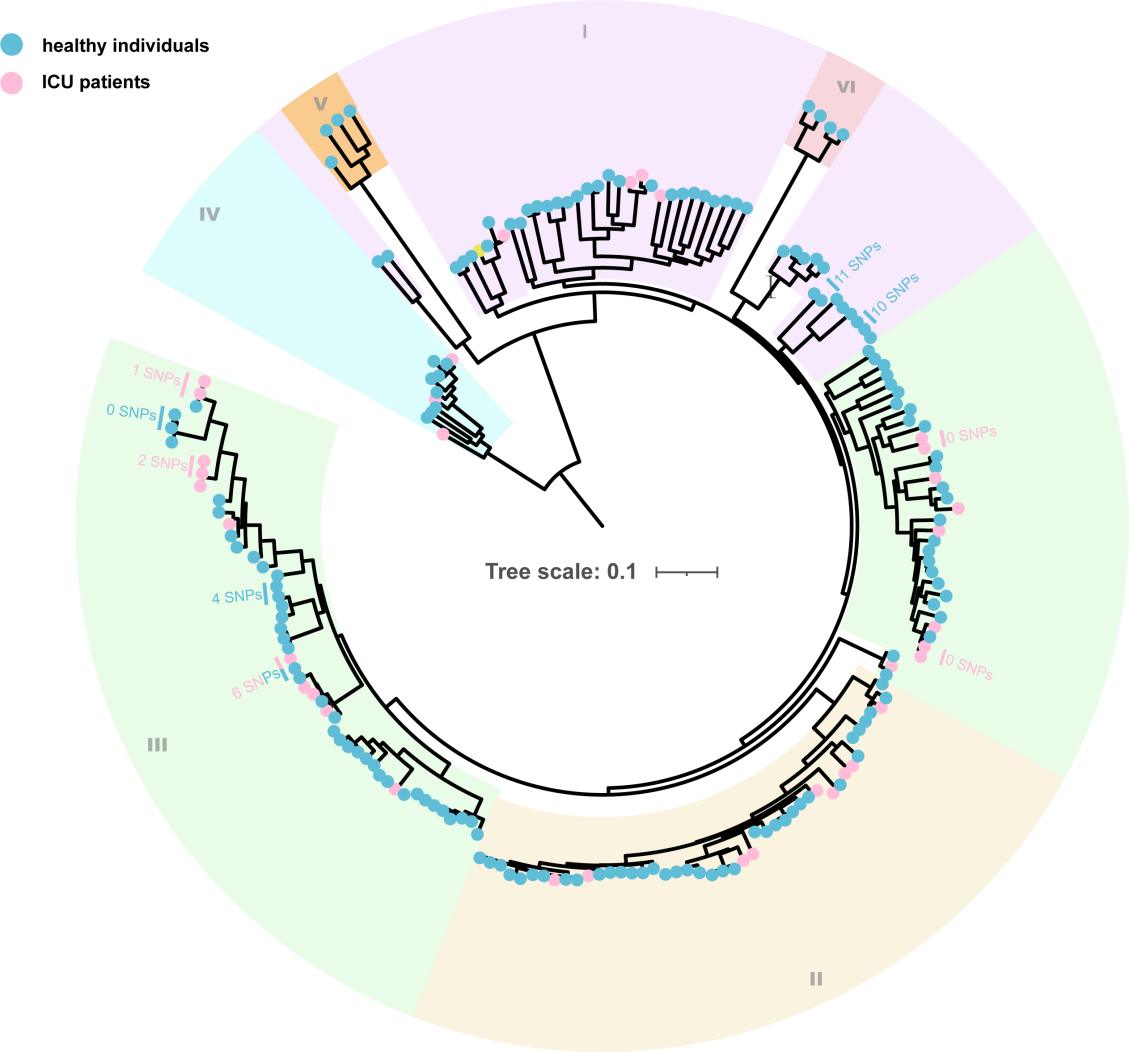
Population structure of 195 *C*. *perfringens* from healthy individuals and ICU patients. Neighbor-joining tree constructed based on 138,483 core-genomic SNPs. Each isolate is labeled on the node with a colored dot representing its origins. The background of the branches is colored in six colors representing the six lineages (I–VI). Selected strain pairs are indicated by the number of SNP differences between their genomes.

To understand the population structure of *C. perfringens* among healthy individuals and ICU patients, we characterized each subset for phylogenetic diversity using *in silico* multi-locus sequence typing. A total of 142 distinct sequence types (STs) were identified from the 195 *C. perfringens* genomes (Table S1). We observed a high ST diversity (quantified by Simpson’s diversity index) and steep ST accumulation curves in both genomes from healthy individuals (0.99) and ICU patients (0.96). This indicates the highly diverse state of *C. perfringens*, and our data set is far from representative of the whole *C. perfringens* populations. We next identified a total of 138,483 core genome single nucleotide polymorphisms (SNPs) among the 195 *C*. *perfringens* genomes and inferred from these SNPs a neighbor-joining phylogeny (Fig. 2). Bayesian analysis of the core genomic SNPs divided the *C. perfringens* genomes into six distinct phylogenetic lineages (I–VI). The majority of strains (90.26%) were clustered into lineages I (*n* = 45), II (*n* = 46), and III (*n* = 85), with median SNP differences of 10.46% (range: 0.01%–16.15%), 3.96% (range: 0.02%–6.79%), and 8.24% (range: 0%–10.14%), respectively. Despite the high population diversity, we observed a minimal number of SNPs (<20) between *C. perfringens* genomes from the healthy individuals and the ICU patients (Fig. 2), suggesting potential transmission events of *C. perfringens* strains in the clinical environment.

The phylogenetic analysis did not reveal any evolutionary branches among isolates from healthy individuals and ICU patients. Instead, *C. perfringens* from ICU patients and healthy individuals showed a generally even distribution throughout the phylogenetic tree (Fig. 2). This observed pattern aligns with findings from the phylogenetic analysis of *C. perfringens* isolates obtained from both human and animal sources (Fig. S1). The even distribution of *C. perfringens* isolates across human and animal hosts underscores the bacteria’s multi-host nature, highlighting the potential zoonotic infection risk associated with this pathogen. However, it should be noted that the analysis is primarily based on toxinotype A strains, which may limit the conclusions.

### The toxigenic genotype of *C. perfringens*

Given the clinical importance of virulence in *C. perfringens*, we conducted a targeted analysis of all known virulence genes within our genomic data set. Among the sequenced isolates, we identified 13 toxigenic genes (Fig. 1c; Table S1). Our data confirmed that the alpha-toxin-producing gene *plc*, clostripain-producing gene *cloSI*, and neuraminidase-producing gene *nanH* are core chromosomal genes of *C. perfringens*. A further 10 other virulence genes were detected in our collection (Table S1). The distribution of these virulence genes did not vary significantly among lineages I, II, and III. Toxin-based typing classified the 195 *C*. *perfringens* isolates into two types, with the majority (191/195) classified as toxinotype A and the minority (4/195) as toxinotype F. Notably, the four F-type *C. perfringens* isolates carrying the enterotoxin gene *cpe* were identified from ICU patients. In addition to the gene *cpe*, which was exclusively present in ICU patients, there was no statistically significant difference in the carriage of the remaining 12 virulence genes between healthy individuals and ICU patients.

## DISCUSSION

Data remain scarce on this clinically and veterinary-important pathogen. Combining epidemiologic surveillance and genomic analysis, our study provides novel insights into *C. perfringens* colonization in people in China. It is evident from our data that the *C. perfringens* was highly present in both healthy individuals and ICU patients. The observed high genetic diversity among isolates in this single clinical set, particularly among isolates from ICU patients with long-term hospital stays, suggests that the potential infectious risk may primarily arise from the bacteria’s gut colonization rather than clonal transmissions within the clinical setting. However, it is worth noting that the observed small number of core genome SNPs among genomes indicates the potential clonal spread of *C. perfringens* within the clinical setting. In this context, spore contamination and the oxygen tolerance of this anaerobic bacterium should be of concern.

Consistent with previous reports, macrolide, lincosamide, and tetracycline resistance appears to be widespread within *C. perfringens* populations, rendering these antibiotics ineffective for treating *C. perfringens* infections. However, our tested fluoroquinolone and beta-lactam antibiotics remained effective against *C. perfringens* and should be prioritized by clinical physicians for treatment. We did not detect the *cfr* gene, which mediates the PhLOPS_A_ phenotype, including resistance to oxazolidinones, in our isolates. Nevertheless, we observed a particular strain with a consistently high MIC to linezolid, despite our inability to identify any known resistance genes linked to this phenotype. Considering previous reports of oxazolidinone resistance in *Clostridium* spp. isolates from animals and humans, these findings highlight the significance of the observed resistance phenotype in *C. perfringens* toward drugs like linezolid, warranting further attention.

In line with previous reports on human-derived *C. perfringens* strains (13), the majority of strains in our study were classified as toxinotype A, owing to its high prevalence in the environment and the intestinal tracts of humans and animals. However, it is noteworthy that four isolates from ICU patients were toxintyped as F, which is frequently associated with foodborne illnesses in humans due to its highly resistant spores and short doubling time (14). Similar to previous studies, our study did not observe a significant correlation between lineage and toxinotype (15). Nevertheless, it is important to acknowledge that the number and diversity of strains analyzed in our study were insufficient to represent the entire *C. perfringens* population, as indicated by the accumulation curves and the high numbers of toxinotype A strains analyzed. Given the overlooked situation of *C. perfringens* as an opportunistic enteric pathogen, future genomic epidemiological studies should be conducted on a larger scale and encompass strains from diverse sources, preferably in a One Health approach. Such studies will be crucial for guiding public health interventions and addressing the current gaps in our understanding of *C. perfringens*.

## MATERIALS AND METHODS

### Subject enrollment and sample collection

From 22 October 2022 to 28 November 2022, we undertook a cross-sectional study to investigate the prevalence of *C. perfringens* in healthy individuals and ICU patients at the Second Affiliated Hospital of Zhejiang University College of Medicine, Hangzhou, China. Healthy adults who were considered generally healthy during their routine physical examinations consented and were included in the study. Their exclusion criteria were gastroenteritis, pregnancy, and subjects receiving antibiotic treatments in the preceding 30 days. Meanwhile, inpatients who were in the ICU for at least 7 days were included on an informed consent basis from their guardians. In total, we collected 699 stool or rectal swabs from the healthy subjects (*n* = 426) and ICU inpatients (*n* = 273) using the ESwab collection kit (Copan, Brescia, Italy) following the manufacturer’s instructions.

To isolate *C. perfringens* strains from the samples, a small amount of feces or 0.2 mL transport liquid from the ESwab tubes was transferred into a 2 mL centrifuge tube containing 0.5 mL of 50% alcohol and left to stand for 2–3 hours. The suspension in the centrifuge tube was mixed thoroughly and then centrifuged at 6,000 rpm for 2 minutes. The sediment was streaked onto tryptose-sulfite-cycloserine agar (Land Bridge, Beijing, China) for anaerobic incubation at 37°C for 20–24 hours (16). Any suspected colonies were purified by 5% defibrinated sheep blood agar and then confirmed as *C. perfringens* using matrix-assisted laser desorption ionization-time-of-flight mass spectrometry (Bruker Daltonik GmbH, Bremen, Germany). The healthy individuals and ICU patients who tested positive for *C. perfringens* in their fecal samples were defined as cases of intestinal *C. perfringens* carriage.

We downloaded all *C. perfringens* assemblies from the NCBI Pathogen Detection database (https://www.ncbi.nlm.nih.gov/pathogens/isolates/) (as of December 2023, *n* = 1,660) and manually curated each isolate for its origin based on the records of “Host,” “Isolation source,” and “Isolation type,” *C. perfringens* isolates were classified as “human” (*n* = 757) or “animal” (*n* = 423) origins based on any clear indication from the records. In the process of constructing the phylogenetic tree, *C. perfringens* with significantly large genomic differences were excluded. Ultimately, 573 *C*. *perfringens* from humans and 418 *C*. *perfringens* from animals were identified.

### Antimicrobial susceptibility testing

To guide a better clinical antibiotic treatment of *C. perfringens* infections, we conducted Etest of the isolated *C. perfringens* against nine antibiotics that are commonly used in *C. perfringens* infections, including metronidazole, penicillin, amoxicillin, tetracycline, ciprofloxacin, cefoxitin, linezolid, clindamycin, and erythromycin. The Etest was performed in accordance with the guidelines of Clinical and Laboratory Standards Institute (CLSI) documents M11-S9 (17) , and the susceptibility was determined according to CLSI documents M100-S30 (18) for metronidazole, penicillin, tetracycline, cefoxitin, and clindamycin (Due to the lack of recommended breakpoints of *Clostridium perfringens* for erythromycin and ciprofloxacin, the same breakpoint as for clindamycin and fluoroquinolone was applied.). Reference strain *Clostridium perfringens* ATCC 13124 served as the quality control strain.

### Genome sequencing and analysis

The genomic DNA of *C. perfringens* isolates was extracted using a PureLink Genomic DNA mini kit (Invitrogen, Carlsbad, CA, USA). Indexed DNA libraries were prepared using a TruSeq DNA PCR-Free Sample Preparation Kit (Illumina, Inc., San Diego, CA, USA), and 300 bp paired-end reads with a minimum of 150-fold coverage for each isolate were obtained following sequencing using the Illumina HiSeq X Ten System. Raw reads were processed by trimming and assembling into contigs using SPAdes version 3.11.1 (19). Given the clinical importance of antimicrobial resistance (AMR) and virulence in *C. perfringens*, a targeted analysis of acquired AMR genes and virulence-factor-associated genes was performed using ABRicate (https://github.com/tseemann/abricate) against the ResFinderFG v2.0 (20), PlasmidFinder (21) database, and VFDB database (http://www.mgc.ac.cn/VFs/) (>90% identity and >75% coverage) (22). Heatmaps describing the prevalence of genes associated with AMR and virulence, as well as resistance phenotypes, were generated using TBtools (23). The genome of one *C. perfringens* isolate that exhibited linezolid resistance was further probed for potential mutations and acquired resistance genes encoding linezolid resistance using LRE-Finder 1.0 (https://cge.food.dtu.dk/services/LRE-finder/).

### Phylogenetic analysis

A core-genome SNP-based neighbor-joining phylogenetic tree was constructed for all sequenced isolates using Parsnp in the Harvest package (24) with default parameter settings. The tree was midpoint-rooted and annotated using iTOL v6 (25). We clustered the sequenced *C. perfringens* strains into several population structures using the Bayesian model-based algorithm, RhierBAPS (26).

### Statistical analysis

Clinical information of healthy individuals and ICU patients was collected via the hospital information system. Differences in each genome’s total number of AMR genes and virulence genes among healthy individuals and ICU patients were assessed using the Wilcoxon test. The Pearson chi-square (χ^2^) test and Fisher’s exact test were used to test whether the differences in frequencies of individual genes encoding resistance or virulence and drug resistance phenotypes were significant. *P*-value < 0.01 was considered significant.

## Data Availability

Data supporting the findings of this study are included in this article and in the supplemental material. Genome assemblies of the *C. perfringens* have been deposited in the NCBI and are registered under BioProject accession no. PRJNA983292. All data are available from the corresponding authors upon reasonable request.

## SUPPLEMENTAL MATERIAL

Fig. S1 spectrum.03385-23-s0001.tif This observed pattern aligns with findings from the phylogenetic analysis of C. perfringens isolates.

Supplemental legends spectrum.03385-23-s0002.docx Legends for Fig. S1 and Table S1.

Table S1 spectrum.03385-23-s0003.xlsx Source, MLST, lineage, SNPs, minimum inhibitory concentration (MIC), plasmid, antibiotic resistance and virulence genes.

ASM does not own the copyrights to Supplemental Material that may be linked to, or accessed through, an article. The authors have granted ASM a non-exclusive, world-wide license to publish the Supplemental Material files. Please contact the corresponding author directly for reuse.
