# Supplementary figures and images for: High intestinal carriage of Clostridium perfringens in healthy individuals and ICU patients in Hangzhou, China
Source: Microbiol Spectr. 2024 May 21;12(7):e03385-23. doi: 10.1128/spectrum.03385-23 (PMC11218483; doi:10.1128/spectrum.03385-23)

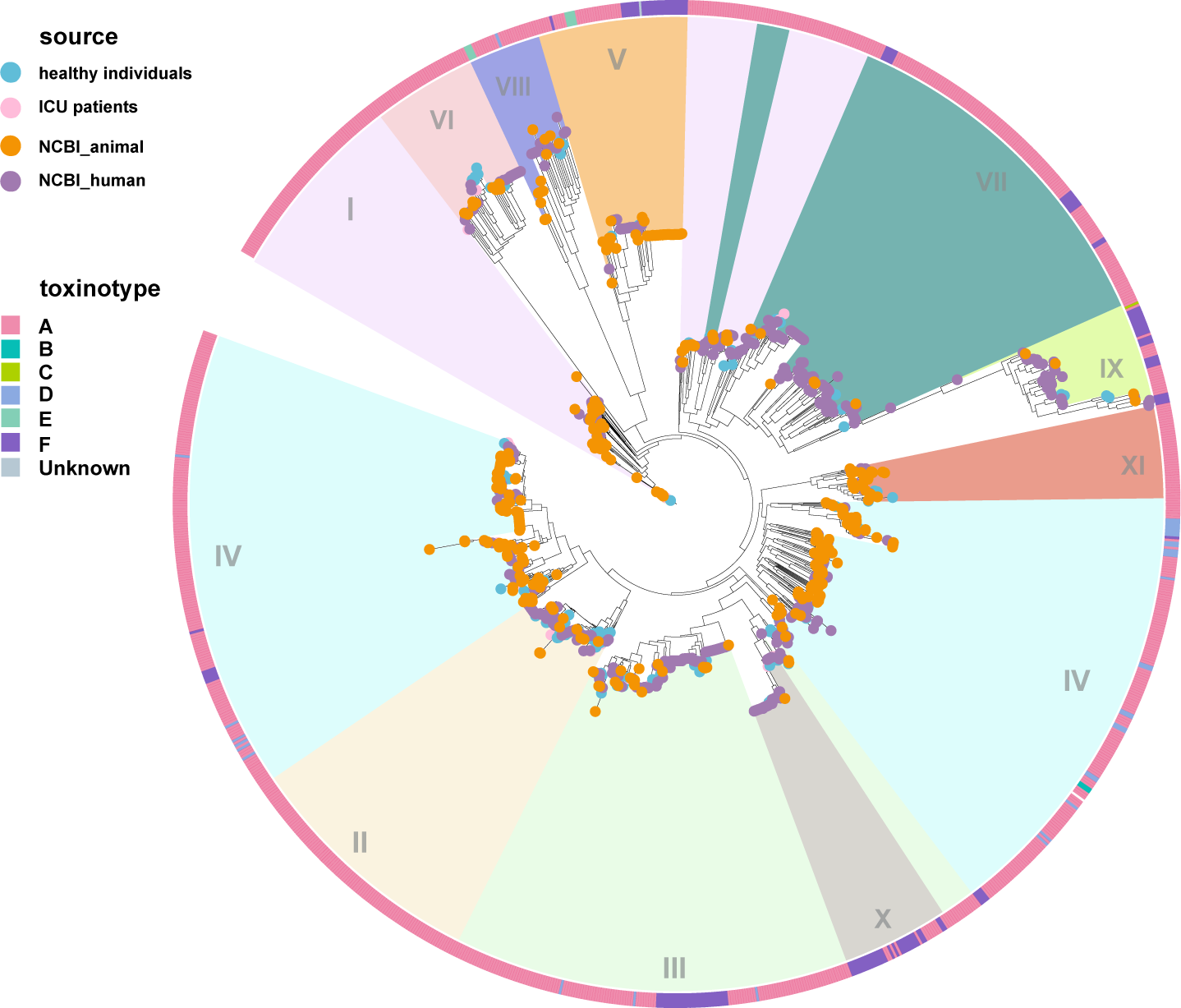

Supplement: Fig. S1 — This observed pattern aligns with findings from the phylogenetic analysis of C. perfringens isolates. [file spectrum.03385-23-s0001.tif]
