# Supplementary material for: High intestinal carriage of Clostridium perfringens in healthy individuals and ICU patients in Hangzhou, China
Source: Microbiol Spectr. 2024 May 21;12(7):e03385-23. doi: 10.1128/spectrum.03385-23 (PMC11218483; doi:10.1128/spectrum.03385-23)
Supplement: Supplemental legends — Legends for Fig. S1 and Table S1. [file spectrum.03385-23-s0002.docx]

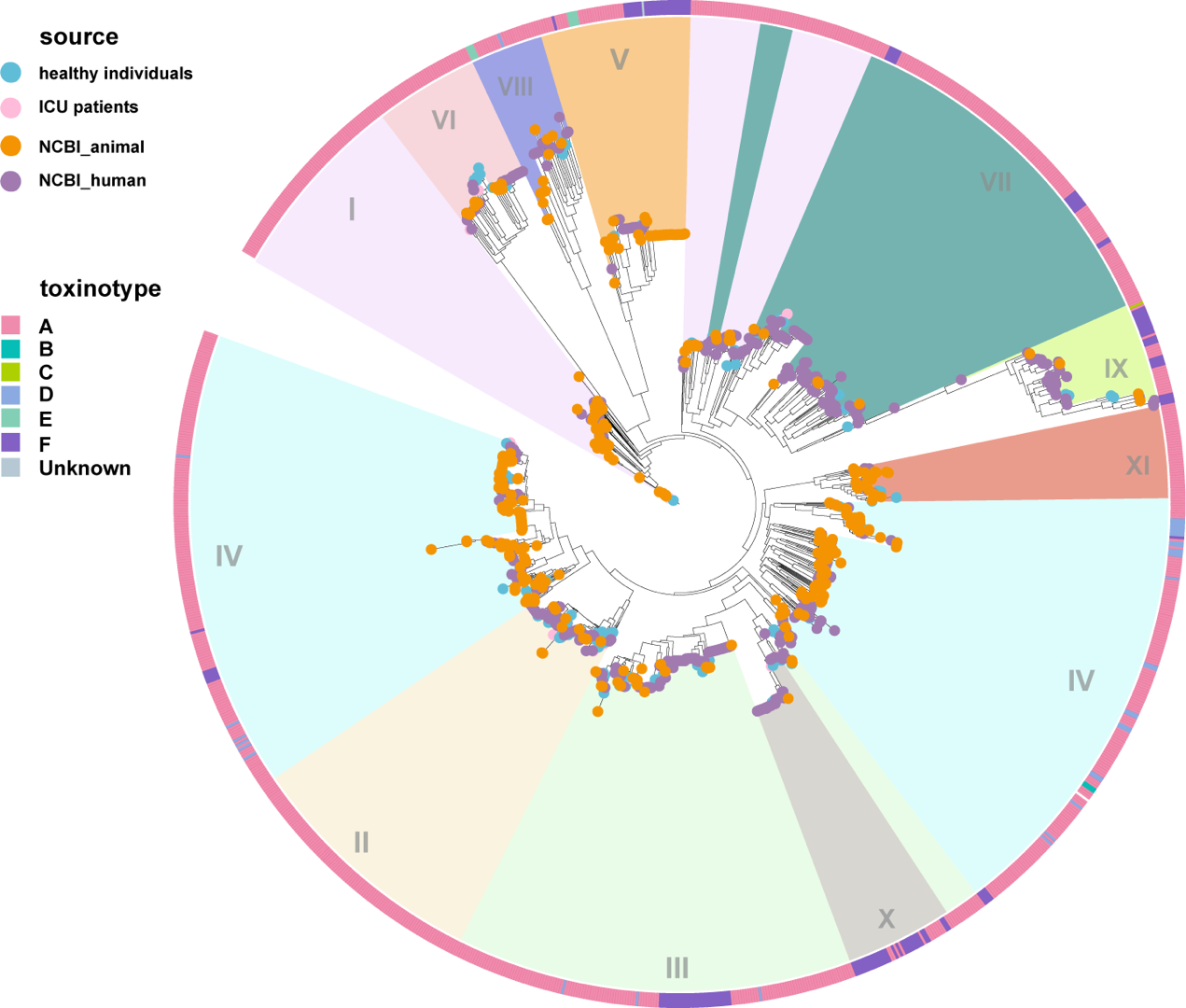


Figure S1: Population structure of 573 human-derived *C. perfringens* and 418 animal-derived *C. perfringens* from NCBI database and 195 *C. perfringens* in this study. The colors and numbers of the ranges represent different population structures based on the Bayesian model. Each isolate is labelled on the node with a colored dot representing its origins. The background of the branches is colored in six colors representing the six lineages (I – VI). The toxinotypes are labelled and shown in the colorful ring.

Table S1: Source, MLST, lineage, SNPs, minimum inhibitory concentration (MIC), plasmid, antibiotic resistance and virulence genes of 195 *C. perfringens*
